# Cue to Acid-Induced Long-Range Conformational Changes in an Antibody Preceding Aggregation: The Structural Origins of the Subpeaks in Kratky Plots of Small-Angle X-ray Scattering

**DOI:** 10.3390/ijms241512042

**Published:** 2023-07-27

**Authors:** Hiroshi Imamura, Shinya Honda

**Affiliations:** 1 Department of Bio-Science, Nagahama Institute of Bio-Science and Technology, 1266 Tamura, Nagahama 526-0829, Japan; 2 Biomedical Research Institute, National Institute of Advanced Industrial Science and Technology (AIST), 1-1-1 Higashi, Tsukuba 305-8566, Japan

**Keywords:** small-angle X-ray scattering, antibody, Kratky analysis

## Abstract

Antibody aggregation, followed by acid denaturation and neutralization of pH, is one of the reasons why the production of therapeutic monoclonal antibodies (mAbs) is expensive. Determining the structural details of acid-denatured antibodies is important for understanding their aggregation mechanism and for antibody engineering. Recent research has shown that monoclonal antibodies of human/humanized immunoglobulin G1 (IgG1) become smaller globules at pH 2 compared to their native structure at pH 7. This acid-denatured species is unstable at pH 7 and prone to aggregation by neutralization of pH. Small-angle X-ray scattering (SAXS) data have revealed an acid-induced reduction in the subpeaks in Kratky plot, indicating conformational changes that can lead to aggregation. The subpeaks are well resolved at pH > 3 but less pronounced at pH ≤ 2. One of the weakened subpeaks indicates loosely organized inter-region (Fab-Fab and Fab-Fc) correlations due to acid denaturation. However, the structural origin of the other subpeak (called *q*_3_ peak in this study) has not been established because its *q* region could represent the various inter-region, inter-domain, and intra-domain correlations in IgG1. In this study, we aimed to untangle the effects of domain–domain correlations on Kratky’s *q*_3_ peak based on the computed SAXS of the crystal structure of IgG1. The *q*_3_ peak appeared in the static structure and was more prominent in the Fc region than in the Fab or isolated domains. Further brute-force analysis indicated that longer domain–domain correlations, including the inter-region, also positively contribute to Kratky’s *q*_3_ peak. Thus, the distortion of the Fc region and a longer inter-region correlation initiate acid denaturation and aggregation.

## 1. Introduction

Protein denaturation and aggregation are inherent and universal phenomena in vivo and ex vivo, and both are often undesirable factors for health. The subject of this study is the latter, ex vivo denaturation and aggregation of therapeutic monoclonal antibodies (mAbs), which are biopharmaceuticals. Because the aggregates of mAbs lead to reduced drug efficacy and potential side effects [1,2], they must be removed during the manufacturing process [3] and further evaluated prior to administration [4], which is time-consuming and costly.

mAbs are purified by affinity chromatography, where an acid solution is used as an eluent [5]. An acidic pH is kept for the subsequent virus inactivation process. During these processes, mAbs are denatured at the acidic pH. The neutralization of pH (pH shift) transforms the denatured mAb molecules into the native structured molecules, but a portion of them associate and evolve to become large aggregates. The mechanism has been identified as “aggregate growth via condensation”, described by Smoluchowski coagulation equations [6]. Unlike amyloid aggregates, the mAb aggregates cannot incorporate the native mAb monomers into the aggregates [7]. Although the aggregate evolution mechanism has been increasingly captured [8], the earlier processes preceding the aggregation remain. Identifying the structural details of the acid-denatured structure is also a necessity for antibody engineering [9].

To address the acid-denatured structure, we have collected small-angle X-ray scattering (SAXS) data of mAbs of human/humanized immunoglobulin G1 (IgG1) in an acid solution [10]. IgG1 is the most popular subclass for therapeutics, which is composed of two identical heavy chains and two identical light chains and is Y-shaped when it is in its native state; the total number of the domains is twelve (cf. Figure 3). Our previous analysis has shown that the structure of the acid-denatured antibody is globular and smaller than the native structure at pH 7 [10]. This anomalous acid-denatured structure is the key structure for the aggregation by the pH shift.

**Figure 1:**
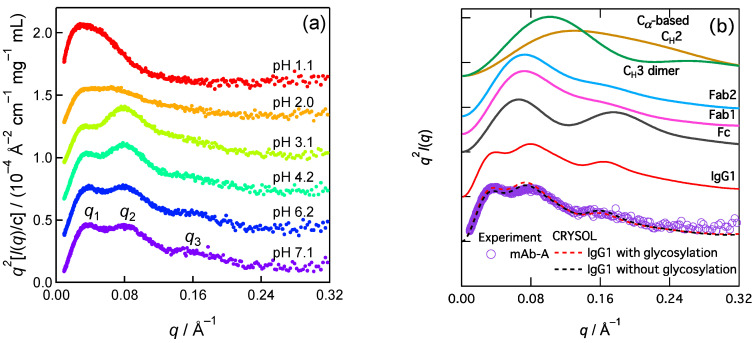
Kratky plots of IgG1 SAXS. (**a**) Experimental results of pH dependence of mAb-A (data taken from ref. [10]). (**b**) computations (solid line: C*_α_*-based method; dashed line: CRYSOL [25] version 2.8.3). In addition to IgG1 (PDB ID: 1HZH), its Fc region, Fab region (1 and 2, see Figure 3), the C_H_3 dimer, and the isolated domain C_H_2 were applied to the SAXS computation. Reprinted (adapted) with permission from ref. [10]. Copyright 2023 American Chemical Society.

The SAXS method is superior for capturing domain–domain correlations (orientation or distance) in multidomain proteins [11], in addition to size information, such as radius gyration (*R*_g_). The SAXS signals of the domain–domain correlations often appear at the *q*-region higher than the Guinier region [12], which should be pronounced as subpeaks in a Kratky plot. Thus, Kratky plot analysis is an important tool for SAXS studies. Our SAXS data depicted the acid-induced reduction in the peaks in the Kratky plot of IgG1, termed mAb-A, in this manuscript (Figure 1a, taken from ref [10]). However, the assignment of the Kratky plot’s subpeaks is nontrivial owing to the various inter-region, inter-domain, and intra-domain correlations in IgG1. In this study, we computed SAXS based on the crystal structure of a representative IgG1 structure and analyzed the effects of the domain–domain correlation on the Kratky plot’s subpeak.

## 2. Results and Discussion

Figure 1a shows the pH dependence of SAXS of mAb-A [10]. The Kratky plot (*q*^2^*I*(*q*) vs. *q*) of SAXS for the native structure (pH 7.1) showed three peaks. These peaks were seen in human IgG1 [13], IgG1s (the origins were not described) [14], humanized IgG1 [15,16,17,18,19], and the other IgG subclasses (humanized IgG2 and humanized IgG4 (S241P mutant)) [15,16], and are thus believed to be shared by many types of mAbs [20]. *I*(*q*) is the SAXS intensity. The *q* (scattering parameter) positions of the peaks were designated as *q*_1_, *q*_2_, and *q*_3_, from the smallest angle to the highest angle, respectively. The *q*_1_ peak (~0.034 Å^−1^) at pH 7.1 indicates *R*_g_ = ~51 Å of the scatter IgG1 according to the Guinier relationship *R*_g_ = 3^0.5^/*q*_1_ [11,21]. The Kratky plot’s *q*_2_ and *q*_3_ subpeaks were well resolved at pH > 3 but were less pronounced at pH ≤ 2.

The *q*_2_ peaks (~0.08 Å^−1^) were rationalized [10,22] and acknowledged [20] as inter-region distances, i.e., Fab (antigen-binding fragment)–Fc (crystallizable fragment) regions distance and Fab–Fab regions distance (~80 Å). Considering the Bragg relation, where the correlation length *r*_c_ is 2π/*q*., would help grasp this relationship between *q*_2_ and the inter-region distances. Accordingly, we interpreted that these inter-region correlations were loosely organized based on the less pronounced *q*_2_ peak at pH ≤ 2. However, the structural origin of the *q*_3_ peak has not yet been established. The *q*_3_ peak is useful for identifying the initial conformational change for acid-denaturation-induced aggregation.

Disentangling the origin of SAXS becomes less straightforward in the higher *q* region due to the second peak of the form factor of the scatter or various correlations [23]. Multidomain or oligomeric proteins possess various distances and orientations, and it is nontrivial to determine the key origins underlying scattering. The computation of SAXS based on structural models is often required to elucidate these origins [24].

We computed the SAXS of a representative IgG1 based on its crystal structure (PDB:1HZH), which indicated that the *q*_3_ peak appeared in the static structure (Figure 1b, red line). In this computation, we used the C*_α_* coordinates to approximate the electron coordinates and determined a distance distribution function (*P*(*r*)) called the C*_α_*-based method. *P*(*r*) is converted to *I*(*q*) via a Fourier transformation. *q* is the scattering parameter. A similar peak appeared in the Fc region but was not prominent in the Fab regions, C_H_3 dimer, and C_H_2. C_H_2 is used as a representative of the isolated domains in IgG1 because all domains share an immunoglobulin fold. The lack of a *q*_3_ peak for C_H_2 suggests that the second peaks of the form factors of the isolated domains did not yield the *q*_3_ peak. In addition to the C*_α_*-based method, we computed SAXS using CRYSOL [25], a standard program for the SAXS of proteins with atomic resolution. The resulting Kratky plot also shows a *q*_3_ peak (Figure 1b, dashed line). The computed SAXS of aglycosylated IgG1 (black dashed line) is comparable to that of glycosylated IgG1 (red dashed line) in the neighborhood of *q*_3_. The minor effects of glycosylation allowed us to neglect the sugar components in the current SAXS calculation for convenience. A previous coarse-grained IgG1 model that consisted of 12 spheres reproduced the *q*_3_ character [18], which encouraged us to investigate the domain–domain correlations and allowed us to use the C*_α_*-based model that has a sufficiently high structural resolution for the discussion of the *q*_3_ peak. The hydration layer can modulate the *q*_3_ peak position and intensity; however, these effects are minor.

**Figure 2:**
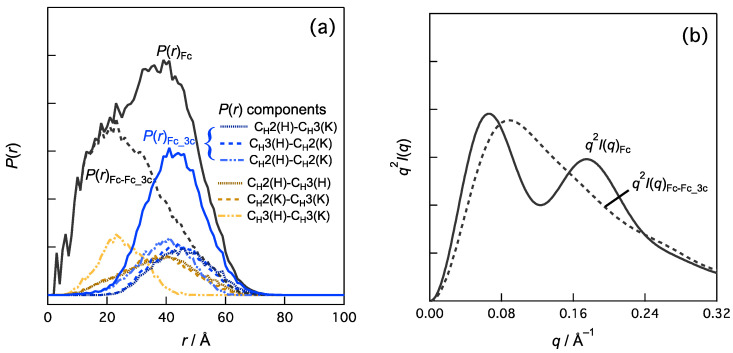
Effects of domain–domain correlations in Fc region on (**a**) *P*(*r*) and (b) *q*^2^*I*(*q*). For example, *P*(*r*)_Fc_3c_ is calculated as follows: *P*(*r*)_Fc_3c_ = *P*(*r*)_CH2(H)–CH3(K)_ + *P*(*r*)_CH3(H)–CH2(K)_ + *P*(*r*)_CH2(H)–CH2(K)_. *I*(*q*)_Fc-Fc_3c_ is given by Fourier transformation of *P*(*r*)_Fc-Fc_3c_ (=*P*(*r*)_Fc_ – *P*(*r*)_Fc_3c_) and represented as Kratky plot in (**b**) (i.e., *q*^2^*I*(*q*)_Fc-Fc_3c_ vs. *q*).

**Figure 3:**
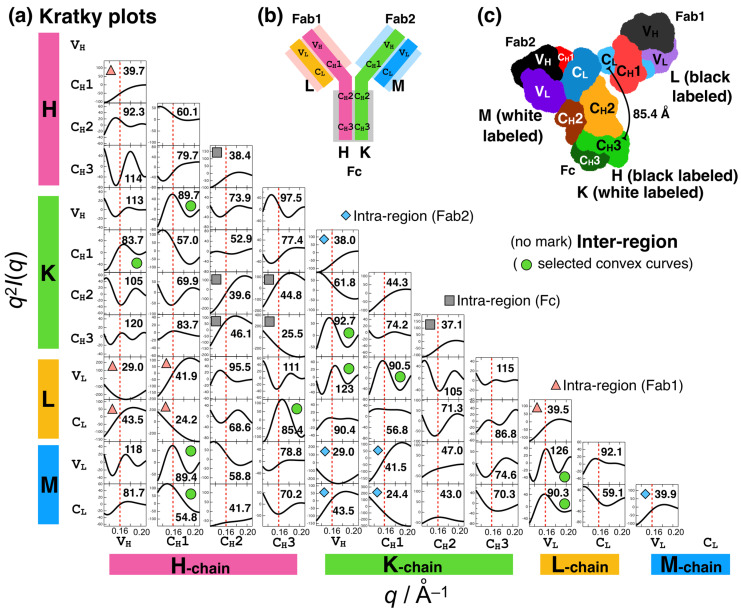
Brute-force analysis of the effects of domain–domain correlations in IgG1 on the Kratky plots. Totally, 66 combinations were calculated. (**a**) Kratky plots for all the domain–domain correlations in IgG1. The gray square, light pink triangle, and blue diamond symbols represent the domain–domain correlations for the intra-regions (Fc, Fab1, and Fab2 regions, respectively). The remains are the domain–domain correlations for the inter-region (e.g., between C_H_3(H) in Fc and C_L_(L) in Fab1). The values in the plots are the averaged distances between the domains. (**b**) Schematic representation of IgG1, its regions (Fc, Fab1, and Fab2), and its domains. Nomenclature of the IgG1 chains (H, K, L, and M) are shown. (**c**) Three-dimensional structure of the IgG1 (PDB:1HZH) used for the present analyses.

A previous SAXS study of Fc [26] proved that the *q*_3_ peak is sensitive to the distances and orientations between the C_H_2 domains and between the C_H_2 and C_H_3 domains in Fc. Fc has two identical chains, referred to as the H-chain and K-chain, according to the annotation in the Protein Data Bank file (PDB ID:1HZH). Each chain had C_H_2 and C_H_3 domains (Figure 3), resulting in six domain–domain correlations. Let us consider the contribution of the domain–domain correlation to the *q*_3_ peak. One domain–domain correlation in real space is represented as the distance distribution component for *P*(*r*) between two domains. We calculated *P*(*r*) of the Fc and *P*(*r*) components stemming from these domain–domain correlations (Figure 2a). The order of the average distances between the domains is as follows:

C_H_2(H)–C_H_3(K) (46.1 Å) > C_H_3(H)–C_H_2(K) (44.8 Å) > C_H_2(H)–C_H_2(K) (39.6 Å)

>C_H_2(H)–C_H_3(H) (38.4 Å) > C_H_2(K)–C_H_3(K) (37.1 Å) > C_H_3(H)–C_H_3(K) (25.5 Å),

where the values in parentheses indicate average distances. The contributions of the top half (the three components in Fc, denoted as Fc_3c), *P*(*r*)_Fc_3c_, were subtracted from the *P*(*r*) values of Fc and *P*(*r*)_Fc_. The reasons for this selection are shown in detail later (Figure 3a). The remaining *P*(*r*)_Fc-Fc_3c_ was calculated as *P*(*r*)_Fc-Fc_3c_ = *P*(*r*)_Fc_ − *P*(*r*)_Fc_3c_ shown in Figure 2a. Consider the following relationship between *P*(*r*) and *I*(*q*).

*I*(*q*) = *FT*(Σ*P*(*r*)*_i_*) = Σ*FT*(*P*(*r*)*_i_*) = Σ*I*(*q*)*_i_*, (1)

where *FT* indicates the Fourier transformation, and the subscript *i* is a component, and we can analyze the effects of *P*(*r*)*_i_* or *I*(*q*)*_i_* on *I*(*q*). Figure 2b demonstrates that the *q*_3_ peak is diminished in the Kratky plot of *I*(*q*)_Fc-Fc_3c_. This indicates that the domain–domain correlations of *P*(*r*)_Fc_3c_ (i.e., C_H_2(H)–C_H_3(K), C_H_3(H)–C_H_2(K), and C_H_2(H)–C_H_2(K)) were responsible for the *q*_3_ peak.

Figure 3 shows all the domain–domain correlations in IgG1, where the Kratky components were computed. For example, the convex curve in the Kratky plot for C_H_2(H)–C_H_3(K) was obtained via Fourier transformation of *P*(*r*) in Figure 2a (blue dotted line). The components in the intra-regions (Fc, gray; Fab1, light pink; and Fab2, light blue) as well as those in the inter-regions are convex curves in the *q*_3_ region and thus positively contribute to the *q*_3_ peak. The Kratky components (10 components, denoted as 10c) indicated by green symbols are stronger convex curves. The average distances between the domains (values in Figure 3a) were ~80–90 and ~125 Å. This is physically reasonable because Bragg’s law is *nλ* = 2*d*sin*θ* (*n* = 1, 2, 3, …). However, assigning which domain–domain correlation is responsible is nontrivial.

**Figure 4:**
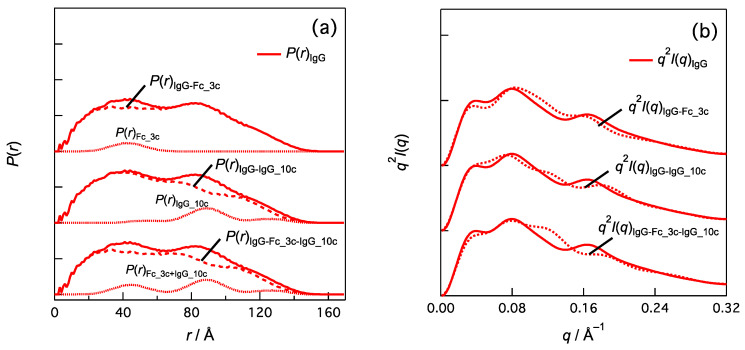
Effects of domain–domain correlations in IgG1 (PDB:1HZH) on (**a**) *P*(*r*) and (**b**) *q*^2^*I*(*q*). For example, *P*(*r*)_IgG_10c_ is calculated by summing *P*(*r*) of the domain-domain correlation indicated by green symbol in Figure 3. *I*(*q*)_IgG-Fc_3c-IgG_10c_ is given by Fourier transformation of *P*(*r*)_IgG-Fc_3c-IgG_10c_ (= *P*(*r*)_IgG_ − *P*(*r*)_Fc_3c_ − *P*(*r*)_IgG_10c_) and represented as Kratky plot in (**b**) (i.e., *q*^2^*I*(*q*)_IgG-Fc_3c-IgG_10c_ vs. *q*).

In Figure 4, subtracting *P*(*r*)_Fc_3c_ from the *P*(*r*) of IgG1 [*P*(*r*)_IgG_] yields *P*(*r*)_IgG-Fc_3c_ and their corresponding Kratky plots indicate the *q*_3_ peak is attenuated and conserved. Subtracting the *P*(*r*) component of 10c [*P*(*r*)_IgG_10c_] from *P*(*r*)_IgG_, which is *P*(*r*)_IgG-IgG_10c_, reduces the *q*_3_ peak intensity and shifts it to a higher *q*. We found that subtracting both [*P*(*r*)_Fc_3c_ and *P*(*r*)_IgG_10c_] effectively diminished the *q*_3_ peak. Other domain–domain correlations and their combinations also contributed to the *q*_3_ peak.

In summary, the origin of the subpeaks in the Kratky plot of IgG1, especially *q*_3_ = ~0.16 Å^−1^, was investigated based on the crystal structure. In addition to the shorter C_H_2-C_H_3 correlations in Fc, the longer correlations between the domains with distances of ~80–90 and ~125 Å also strongly contributed to this *q*_3_ peak. For example, C_H_3(H) in Fc and C_L_(L) in Fab1 are separated at an averaged distance of 85.4 Å and this combination is the positive contributor. The contributions of the domain–domain correlations are shown in Figure 3. The acid-induced conformational change in IgG1 that triggers aggregation includes the loss of these longer domain–domain correlations that are stable in the native state.

In this study, we used the single static structure of IgG1 while the domain–domain correlation of IgG1 can be stochastic; this is the limitation of this study. Thus, the effects of structural dynamics should be noted. A previous study [26] identified that the position and intensity of the *q*_3_ peak are sensitive to conformational fluctuations (i.e., open–close or C_H_2 domain orientation) in the Fc region. A specific combination positively (or negatively) contributing to the *q*_3_ peak in this analysis (Figure 3a) could become a negative (or positive) contributor due to changes in the distance distribution caused by conformational fluctuations in IgG1.

The subpeaks in the Kratky plots involve many correlations. Assigning them should be in conjunction with *I*(*q*) analysis at lower *q* to higher *q* and *P*(*r*) analysis at smaller *r* to longer *r* to be self-consistent. Such an analysis would be more feasible for a monodispersed sample (impurity-free or aggregate-free) and for SAXS data with a higher S/N ratio. Even if the mixture contains unavoidable large impurities, such as aggregates, the SAXS of the large particles decays steeply and is less prominent in the higher *q* region, in which Kratky analysis is preferred, and extracts information from such limited data. Even if the preliminary data are noisy, the Kratky peak is useful for deducing structural changes, which can serve as a working hypothesis for further research.

Interestingly, this *q*_3_ peak is shared by IgG1, IgG2, and IgG4 and is less pronounced upon heat-induced aggregation (IgG1) [17] or acid-induced aggregation (IgG2 and IgG4) [15]. The long-range conformational changes identified in this study are the initial common events that precede the aggregation of various mAbs under a wide variety of stresses. The *q*_3_ peak in the Kratky plot can be used as a good indicator of the native-like, long-range-ordered structure or the dynamics of mAbs.

## 3. Materials and Methods

### 3.1. SAXS Computation

The following SAXS calculation is called “C*_α_*-based” computation in this manuscript. The measure of the distance between selected pairs of IgG1 C*_α_* coordinates (PDB ID:1HZH) provides the number of pairs of points separated by a distance, *r*, termed *n*(*r*). *n*(*r*) is proportional to the distance distribution function *P*(*r*) of the SAS object and is therefore regarded as *P*(*r*). Scattering *I*(*q*) was calculated using the Fourier transform of *P*(*r*), as shown in Equation (2):

(2) $$I(q)=4\pi \int_{0}^{D_{\max}}P(r)\frac{\sin(qr)}{qr}dr$$

where *q* is defined as *q* = |***q***| = 4πsin*θ*/*λ*, ***q*** is the scattering vector, 2*θ* is the scattering angle, and *λ* is the wavelength of the X-ray or neutron. *n*(*r*), *P*(*r*), and *I*(*q*) were processed using an in-house program [22] in IGOR Pro version 6.22A (WaveMetrics, Portland, OR, USA).

Each domain of IgG1 was assigned according to “Family & Domains” information in UniProt (https://www.uniprot.org (accessed on 25 July 2023)) after sequence alignments. The UniProt IDs used were P01857 (C_H_1), P01857 (C_H_2), P01857 (C_H_3), P0CG04 (C_L_), P01825 (V_H_), and P01703 (V_L_). Linker regions were not included in the *I*(*q*) calculation.

This study focused on extracting the inter-domain correlations, for which “C*_α_*-based” calculation based on Equations (1) and (2) is straightforward. The CRYSOL program [25] outputs *I*(*q*), which involves both the intradomain and the inter-domain correlations, and thus does not intend to address the decomposition of these contributions.

### 3.2. SAXS Data Collection

Experimental SAXS data for humanized immunoglobulin G1 (IgG1) (148 kDa), termed mAb-A, were collected using the BL-10C beamline at the Photon Factory of the High Energy Accelerator Research Organization (KEK) in Tsukuba, Japan [27]. The data of Figure 1a were obtained from Figure 1b in the literature [10]. The mAb-A sequence was almost identical to that of the representative IgG1 model (PDB ID:1HZH), except for the V_H_ and V_L_ sequences.

## Data Availability

Data presented in this study are available in the manuscript. The data generated and/or analyzed during the current study are available from the corresponding author upon reasonable request.

## References

[1] Volkin D.B., Hershenson S., Ho R.J.Y., Uchiyama S., Winter G., Carpenter J.F. (2015). Two Decades of Publishing Excellence in Pharmaceutical Biotechnology. J. Pharm. Sci..

[2] Moussa E.M., Panchal J.P., Moorthy B.S., Blum J.S., Joubert M.K., Narhi L.O., Topp E.M. (2016). Immunogenicity of Therapeutic Protein Aggregates. J. Pharm. Sci..

[3] Vázquez-Rey M., Lang D.A. (2011). Aggregates in Monoclonal Antibody Manufacturing Processes. Biotechnol. Bioeng..

[4] Jiskoot W., Nejadnik M.R., Sediq A.S. (2017). Potential Issues with the Handling of Biologicals in a Hospital. J. Pharm. Sci..

[5] Imamura H., Honda S. (2019). pH-Shift Stress on Antibodies. Methods Enzymol..

[6] Imamura H., Honda S. (2016). Kinetics of Antibody Aggregation at Neutral pH and Ambient Temperatures Triggered by Temporal Exposure to Acid. J. Phys. Chem. B.

[7] Imamura H., Sasaki A., Honda S. (2017). Fate of a Stressed Therapeutic Antibody Tracked by Fluorescence Correlation Spectroscopy: Folded Monomers Survive Aggregation. J. Phys. Chem. B.

[8] Senga Y., Imamura H., Ogura T., Honda S. (2019). In-Solution Microscopic Imaging of Fractal Aggregates of a Stressed Therapeutic Antibody. Anal. Chem..

[9] Hebditch M., Kean R., Warwicker J. (2020). Modelling of pH-Dependence to Develop a Strategy for Stabilising MAbs at Acidic Steps in production. Comput. Struct. Biotechnol. J..

[10] Imamura H., Ooishi A., Honda S. (2023). Getting Smaller by Denaturation: Acid-Induced Compaction of Antibodies. J. Phys. Chem. Lett..

[11] Svergun D.I., Koch M.H.J., Timmins P.A., May R.P. (2013). Small Angle X-ray and Neutron Scattering from Solutions of Biological Macromolecules.

[12] Fujisawa T.T., Inoko Y., Kataoka M. (1987). X-ray Scattering from a Troponin C Solution and Its Interpretation with a Dumbbell-Shaped-Molecule Model. J. Appl. Cryst..

[13] Ashish, Solanki A.K., Boone C.D., Krueger J.K. (2010). Global Structure of HIV-1 Neutralizing Antibody IgG1 b12 is Asymmetric. Biochem. Biophys. Res. Commun..

[14] Lilyestrom W.G., Shire S.J., Scherer T.M. (2012). Influence of the Cosolute Environment on IgG Solution Structure Analyzed by Small-Angle X-ray Scattering. J. Phys. Chem. B.

[15] Tian X., Langkilde A.E., Thorolfsson M., Rasmussen H.B., Vestergaard B. (2014). Small-Angle X-ray Scattering Screening Complements Conventional Biophysical Analysis: Comparative Structural and Biophysical Analysis of Monoclonal Antibodies IgG1, IgG2, and IgG4. J. Pharm. Sci..

[16] Tian X., Vestergaard B., Thorolfsson M., Yang Z., Rasmussen H.B., Langkilde A.E. (2015). In-Depth Analysis of Subclass-Specific Conformational Preferences of IgG Antibodies. IUCrJ.

[17] Barnett G.V., Razinkov V.I., Kerwin B.A., Laue T.M., Woodka A.H., Butler P.D., Perevozchikova T., Roberts C.J. (2015). Specific-Ion Effects on the Aggregation Mechanisms and Protein-Protein Interactions for Anti-Streptavidin Immunoglobulin Gamma-1. J. Phys. Chem. B.

[18] Godfrin P.D., Zarraga I.E., Zarzar J., Porcar L., Falus P., Wagner N.J., Liu Y. (2016). Effect of Hierarchical Cluster Formation on the Viscosity of Concentrated Monoclonal Antibody Formulations Studied by Neutron Scattering. J. Phys. Chem. B.

[19] Castellanos M.M., Howell S.C., Gallagher D.T., Curtis J.E. (2018). Characterization of the NISTmAb Reference Material using Small-Angle Scattering and Molecular Simulation: Part I: Dilute Protein Solutions. Anal. Bioanal. Chem..

[20] Wang T., Chen J., Du X., Feng G., Dai T., Li X., Liu D. (2022). How Neutron Scattering Techniques Benefit Investigating Structures and Dynamics of Monoclonal Antibody. Biochim. Biophys. Acta Gen. Subj..

[21] Semisotnov G.V., Kihara H., Kotova N.V., Kimura K., Amemiya Y., Wakabayashi K., Serdyuk I.N., Timchenko A.A., Chiba K., Nikaido K. (1996). Protein Globularization During Folding. A Study by Synchrotron Small-angle X-ray Scattering. J. Mol. Biol..

[22] Imamura H. (2023). Phone2SAS: 3D Scanning by Smartphone Aids the Realization of Small-Angle Scattering. Biophys. Physicobiol..

[23] Phan-Xuan T., Bogdanova E., Millqvist Fureby A., Fransson J., Terry A.E., Kocherbitov V. (2020). Hydration-Induced Structural Changes in the Solid State of Protein: A SAXS/WAXS Study on Lysozyme. Mol. Pharm..

[24] Zhang F., Roosen-Runge F., Sauter A., May R.P., Skoda M.W.A., Jacobs R.M.J., Sztucki M., Schreiber F. (2012). The Role of Cluster Formation and Metastable Liquid—Liquid Phase Separation in Protein Crystallization. Faraday Discuss..

[25] Svergun D., Barberato C., Koch M.H.J. (1995). CRYSOL—A Program to Evaluate X-ray Solution Scattering of Biological Macromolecules from Atomic Coordinates. J. Appl. Crystallogr..

[26] Yageta S., Imamura H., Shibuya R., Honda S. (2019). C_H_2 Domain Orientation of Human Immunoglobulin G in Solution: Structural Comparison of Glycosylated and Aglycosylated Fc Regions Using Small-Angle X-Ray Scattering. MAbs.

[27] Shimizu N., Mori T., Nagatani Y., Ohta H., Saijo S., Takagi H., Takahashi M., Yatabe K., Kosuge T., Igarashi N. (2019). BL-10C, the Small-Angle X-Ray Scattering Beamline at the Photon Factory. AIP Conf. Proc..

